# Patients with dysphagia: How to supply nutrition through non-tube feeding

**DOI:** 10.3389/fnut.2022.1060630

**Published:** 2022-12-02

**Authors:** Zhean Shen, Yingze Hou, Ayideng Huerman, Aiqin Ma

**Affiliations:** ^1^College of Food Science and Engineering, Xinjiang Institute of Technology, Aksu, China; ^2^Nutritional Department, Shanghai Jiao Tong University Affiliated Sixth People's Hospital South Campus, Shanghai, China; ^3^Sanquan College, Xinxiang Medical University, Xinxiang, China

**Keywords:** dysphagia, texture-modified foods, swallowing fluid mechanics, non-tube feeding, thickened food

## Abstract

**Objective:**

Dysphagia has become one of the important factors that cause malnutrition in the whole age group. At present, tube feeding is still the mainstream means to solve the problem of dysphagia. However, tube feeding has physical and mental harm to people, and the ways of non-tube feeding are relatively diversified. The significance of the thickening mechanism described in some articles to solve the problem of dysphagia is not clear.

**Setting and participants:**

All patients with dysphagia worldwide, including oropharyngeal dysphagia (OD) and non-oropharyngeal dysphagia.

**Methods:**

We searched the literature in Pubmed, Web of Science and Cochrane Library and initially browsed the titles and abstracts. We reviewed the full text of the articles that met our topic, and the language of the article was limited to English.

**Results:**

We found that food thickening to a certain degree (350–1,750 cP) can reduce the complications of choking, aspiration, reflux, and other complications in patients with dysphagia, and reduce the social disorder, anxiety, and other psychological problems caused by catheterization and surgery. Significantly, food science engineers should invite clinicians to intervene in the development of specialty foods from different perspectives such as clinical pathophysiology and fluid mechanics.

**Conclusion and implications:**

It is necessary to develop special foods for patients with dysphagia, which requires scientists from different disciplines to work together.

## Introduction

Swallowing is defined as the function of clearing food and drink through the oral cavity, pharynx, and esophagus into the stomach at an appropriate rate and speed defined by the International Classification of Functioning, Disability and Health (ICF, code b5105) promoted by the World Health Organization (WHO) (1). Dysphagia is classified under “digestive symptoms and signs” in the International Classification of Diseases (ICD-10, code R13), also promoted by WHO (1). Dysphagia is defined as obstruction and stagnation in the pharynx, sternum or xiphoid region resulting from obstruction of food delivery from mouth to stomach or cardia, it has become one of the most important causes of human nutrition intake difficulties (2, 3). Dysphagia is estimated to affect^*^8% of the world's population (^*^590 million people) (4). However, dysphagia is most commonly due to chronic benign disorders, although it can be associated with appreciable morbidity and impaired quality of life (QOL) (5). Besides, consequences of dysphagia include delayed return to oral intake (6, 7), pneumonia, poor quality of life, longer intensive care and hospital stays and is an independent predictor for 90-day mortality (7–12). It remains an under-recognized but highly relevant clinical challenge with symptoms found to persist beyond hospital discharge for >6 months in 23% of patients in a multicentre 5-year longitudinal study (13–16). Nowadays, tube feeding is the mainstream of clinical intervention for dysphagia, but it is easy to bring discomfort and pain to patients, and at the same time, it reduces the quality of life of such groups (9, 10, 12). Not only that, up to 67% of patients intubated for prolonged periods can be affected (17, 18). For example, tube feeding may lead to social embarrassment, lack of food taste and reduced daily activity may have a significant psychological impact on patients themselves or their families and social workers (19). Therefore, dysphagia has been a serious threat to people 's physical and mental health, and there is no reliable non-invasive intervention method. Based on this, we plan to provide food scientists with better quality choices and references by reviewing the advanced and reliable literature on making foods specifically intended for patients with dysphagia. In general, dysphagia caused by diseases is divided into two parts. One is neurogenic dysphagia, such as stroke sequelae, and the other is non-neurogenic dysphagia, such as Iron deficiency dysphagia and multisystem atrophy (2). Therefore, we divide dysphagia into OD and non-OD when searching articles, so that food scientists can develop food more specifically. We used Pubmed, Web of Science, and the Cochrane Library to search for relevant literature. We first scanned the titles and abstracts, then reviewed the full text of the articles that met our topic, and screened them based on the reliability of experimental design and statistical results. The language of the included literature is English only.

## Oropharyngeal dysphagia

### Clinical features of OD

The definition of “oropharyngeal period” in swallowing refers to at the beginning of the involuntary throat period, the soft palate rises and closes the nasopharynx to prevent reflux. Then the hyoid bones rise, the throat rises, and the apnoea protects the throat. At the same time, the tongue base contacted the pharyngeal wall, and the subpharyngeal muscle contracted, the musculus cricopharyngeus relaxed, and the upper esophageal sphincter opened (20, 21). OD includes a group of symptoms and signs which refers to difficulty in forming or moving a bolus safely from the oral cavity to the esophagus (22). A meta-analysis discussed the location of dysphagia and concluded that hypopharyngeal and piriform sinus disease is more common in the elderly or other patients with dysphagia (23). According to WHO, the main symptoms of OD are aspiration, residual, excessive throat clearing, cough, hoarseness, atypical breathing and repeated swallowing (24). The prevalence of dysphagia varies according to potential etiology, age, environment, and source of information. Overall, the prevalence of oropharyngeal dysphagia is between 6 and 50%, while in the unhospitalized elderly population, the prevalence of oropharyngeal dysphagia is 11–16%, while in the physically unwell elderly, the figure is 55% (19, 25). However, OD is a symptom of geriatric syndrome. One study group reported that OD showed higher short-term and long-term related mortality in the elderly with dysphagia (24, 26). OD can bring many complications, such as malnutrition, reflux pharyngitis easy to cause airway foreign body obstruction (19).

There are many reasons for OD, such as epiglottis dysfunction after stroke, oropharyngeal cancer surgery, sarcopenia, or iatrogenic factors. The combination of one or more of the above factors can lead to OD. The older adults often suffer from neurological diseases that cause OD. According to statistics, 64% of stroke patients and more than 80% of dementia patients will have OD (18, 20, 27).

### Existing solutions

Chest infections and malnutrition are often common complications in patients with OD, and reducing the incidence of complications and mortality is often the primary treatment goal for dysphagia (28). For this purpose, we have an endoscopic gastroenteric tube, nasogastric tube, intravenous parenteral nutrition (PN) and other methods. These methods are effective, but also inevitably bring physical and psychological trauma to patients. In adults, for example, randomized controlled trials (RCTs) showed that the nasogastric feeding tube as a risk factor for aspiration pneumonia (29). In a meta-analysis of 82 RCTs, PN was associated with a significantly increased risk of infectious complications (30). In addition, people with OD and who receive nasal feeding are prone to nasopharyngeal congestion, an discomfort that can lead to anxiety (31). According to Singhal et al. (32), tube feeding is generally used in people with oral undernutrition or relatively unsafe oral diets and with gastrointestinal function (Table 1).

**Table 1:** Indications for tube feeding.

1. Insufficient oral intake
- Anorexia
- Food aversion
- Malabsorption (cysticfibrosis, short bowel syndrome, pancreatic insufficiency)
- Increased needs (congenital heart disease, bronchopulmonary dysplasia)
2. As a primary therapy
- Metabolic disease
- Intolerance to fasting
- Inflammatory bowel disease
3. Oral motor dysfunction
- Prematurity
- Neuromuscular disease
- Neurologic disease
4. Abnormal gastrointestinal tract
- Congenital malformations
-Esophageal stenosis
- Intestinal pseudo-obstruction
5. Injury/critical illness
- Burn
- Trauma
- Surgery
- Sepsis

Adapted with permission from: Baker et al. (33).

Percutaneous endoscopic gastrostomy (PEG) is an advanced method to solve malnutrition caused by OD, compared with the method of gastrointestinal tube implantation, it reduces the damage of mumps and nasal cartilage. However, about 13–40% of patients with PEG will have mild complications, such as gastric content leakage around the tube leading to immersion, and perioral pain. It was reported that 0.4–4.4% of the operations had severe complications requiring further intervention, including perioral leakage of peritonitis, necrotizing fasciitis in the anterior abdominal wall, gastric bleeding, visceral injury, tumor spread and death at the PEG site. The mortality rate at 30 d after PEG was reported to be 6.7–26% (30, 34–37). The most terrible complication of PEG is esophageal perforation (0.008–0.04%). Perforation caused by abnormal esophageal anatomy is as high as 50%. Therefore, we still need to find a more advanced solution to solve the malnutrition problem of such patients.

### How to solve OD by non-traumatic method: From the angle of fluid mechanics and food additives

**Figure 1:**
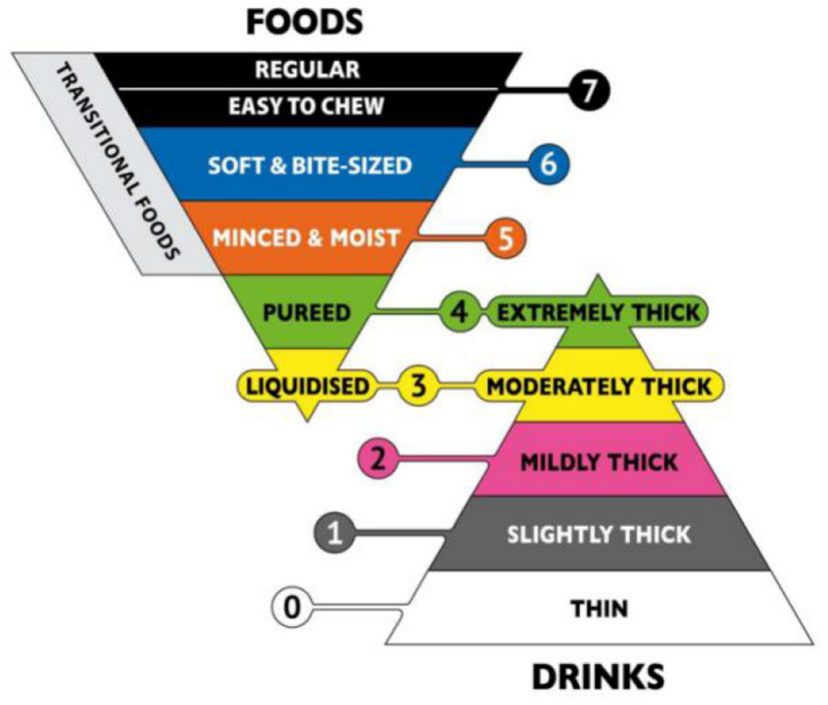
The International Dysphagia Diet Standardization Initiative framework. Reprinted with permission from Hind et al. (39).

**Figure 2:**
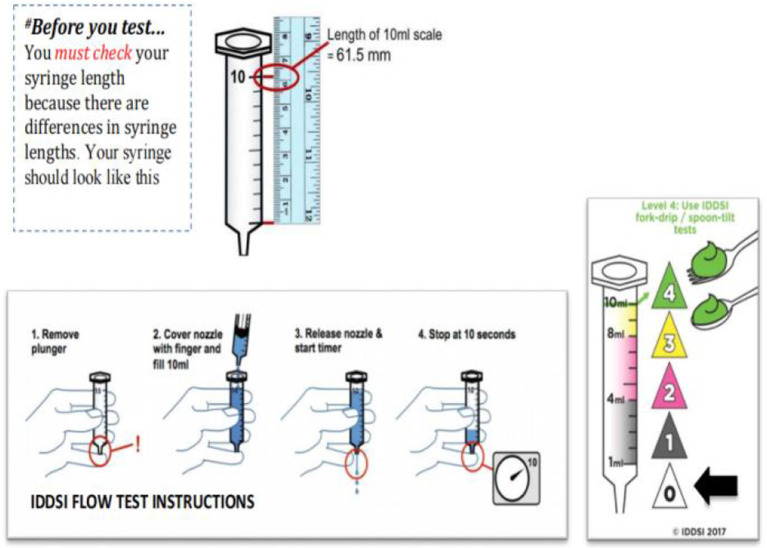
The International Dysphagia Diet Standardization Initiative Flow Test Instructions. Reprinted with permission from Hind et al. (39).

A retrospective case-control study showed that relaxation of upper esophageal sphincter (UES) may lead to incomplete opening and may lead to nasal reflux (38). Therefore, it is necessary to intake texture-modified foods because it can delay the swallowing process of patients and allow the nerve to have enough time to reflect. However, there are different ways to produce this texture-improved food globally, and there is no clear regulation on food traits. In this context, the International Dysphagia Diet Standardization Initiative (IDDSI) released a framework for international standardization terminology and definitions in 2016 and update them in 2019. The framework of IDDSI divides beverages and foods into eight consecutive grades. Beverages are 0–4 (Thin-Extremely thick) and foods are 3–7 (Liquidized-Easy to chew/Regular) (Figure 1) (40). Because the determination of food viscosity and rheological properties requires specialized instruments, such as rheometer, but the use and operation of these machines require operational training and so on, which is not suitable for simple clinical tests (41). Therefore, IDDSI proposes a simple test method for determining the rheological properties of foods. The first step is to remove your plunger, the second step is to cover the nozzle with a finger and fill 10 mL, the third step is to release the nozzle & start the timer, the fourth step is to stop at 10 s (Figure 2) (40).

Due to the decrease of nerve reflex or muscle function in OD patients, it is necessary to focus on the rheological and textural properties, hardness, tensile viscosity of food when preparing food suitable for OD patients (41, 42). A review by Steele et al. pointed out that hardness, cohesiveness and smoothness were important factors for texture modified food (43). Wei et al.'s research proposal is that to achieve safe swallowing, nutrition liquids must be thickened. Thickened fluids Flow slowly, allowing for better oral and pharyngeal coordination, and thus enhance safe swallowing in patients with dysphagia (44). We found four rheological studies on developing food for OD patients. The study of Weston (45) used IDDSI method to carry out the flow test of blending formula (BF). The results showed that the viscosity of BFs changed greatly and could be used for various purposes. The extremely thick formula can be used to help improve oral and gastric fistula in children with gastrointestinal symptoms. BFs with specific thickness can be used as a solution for dysphagia. When BF is used, it may be difficult to recommend a thickening level without knowing the viscosity of the formula. IDDSI test can be used to ensure the necessary thickness is ready (46). In the case of enteral feeding, thinner viscosity may be required, especially if BFs are fed through the enteral feeding pump. Two studies (47, 48) compared flaxseed gum (FG) with commercial thickeners based on xanthan gum (XG) and modified starch (MS). Flow curves were obtained by an up down–up step program using different shear stress ranges to provide shear rates. Newtonian [Eq. (1)], power-law [Eq. (2)] and Herschel-Bulkley [Eq. (3)] equations were fitted to the data to obtain rheological properties:


(1) $$\begin{array}{l} \sigma =\eta \cdot \dot{\gamma } \end{array}$$

(2) $$\begin{array}{l} \sigma =k\cdot {\dot{\gamma }}^{n} \end{array}$$

(3) $$\begin{array}{l} \sigma =\sigma_{0}+k\cdot {\dot{\gamma }}^{n} \end{array}$$

*Notes: Where* σ *is the shear stress (Pa)*, η *is the viscosity (Pa.s), k is the consistency index (Pa*.*s*^*n*^*)*,$\dot{\gamma }$ *is the shear rate (s*^−1^*) and n is the flow index*.

The lowest concentration of XG was similar to the highest concentration of FG η ^*^, indicating that XG had a strong thickening ability. At the same concentration of MS and commercial MS thickening solution, the composite viscosity had no significant difference.

Hind et al. (39) studies have shown that the liquid with shear viscosity (50 *s*^−1^) above 1,500 mPa s is more likely to remain in the swallowing after swallowing. The oral preparation phase may involve lower shear rates (especially for thicker liquids), and thickness perception is most relevant to objective viscosity measurements in 10/s (49). However, it is unclear whether the shear viscosity is the only relevant rheological parameter in this regard.

Hadde et al. (50) studies was carried out experiments on protein to change the tensile viscosity and cohesiveness of food and found that since milk contains biopolymer (protein), the surface tension of concentrated skimmed milk is lower than that of concentrated water (*p* < 0.01). This behavior has a certain guiding significance for the management of dysphagia. If the viscosity of the liquid is affected by the change of surface tension, or is inherent in the liquid, or is due to the addition of thickener, it may affect the ability of pellets to maintain the unit without fracturing during swallowing.

Phase angle (δ) and loss angle tangent (tanδ) are also used to characterize the thickening fluid and express the related aspects of viscous and elastic components. The phase angle represents the phase difference between the applied strain and the measured response in the material. In pure elastic behavior, the phase angle isδ = 0° (Hooke solid), while in pure viscous behavior, the phase angle isδ = 90° (Newtonian fluid). The phase angle between 0°and 90° represents the Viscoelastic floating. The loss angle tangent (tanδ) describes the viscoelastic equilibrium of the material (Eq. 4).


(4) $$\begin{array}{l} \tan\delta =\frac{G^{\prime \prime }}{G^{\prime }} \end{array}$$

Therefore, the tanδ value of the strong gel is below 0.1, that of the dilute solution is above 1, and that of the weak gel is between 0.1 and 1 (41). According to Sharma et al. the higher tanδ value, the higher viscous component (G00) can make the food pellets more easily swallowed (51). For example, banana weak gel has a tanδ value between 0.1 and 0.13, so it is weak gel and within safe swallowing range (52).

In terms of specific food applications (Table 2), Wang et al. (53) conducted a randomized controlled trial on the effect of capsaicin on swallowing function in stroke patients with dysphagia. The results showed that the conventional application of natural capsaicin could promote the recovery of swallowing function in stroke patients with dysphagia. A large number of natural capsaicin can provide a low-cost, easy-to-obtain and safe alternative method to solve the dysphagia of stroke patients. Rofes et al. conducted similar studies. They found that the addition of bi TRPV1/TRPA1 agonist piperine in food could significantly improve the swallowing safety of patients with dysphagia by accelerating the airway closure time, thereby reducing the incidence and severity of penetration and inhalation (54).

**Table 2:** Summary of food products suitable for OD patients.

**Investigator**	**Target population**	**Results**
Wang et al. (53)	OD patients caused by stroke	Natural capsaicin can promote the recovery of swallowing function is such patients and can be considered to be added to food.
Rofes et al. (54)	Elderly OD patients	Adding piperine to food can significantly improve swallowing safety in patients with dysphagia by accelerating airway closure time.
Reyes-Torres et al. (51)	Elderly OD patients	The physiological indexes of patients with nectar viscosity (51–350 cP) or honey viscosity (351–1,750 cP) were better than those with traditional food.
Higashiguchi et al. (55)	Patients with chewing disorder and mild OD	A new type of enzyme-processed softening food may be suitable for OD patients.
Kyodo et al. (56)	Moderate to severe OD patients	Purred diets containing a gelling agent appeared to reduce the risk of aspiration pneumonia possibly by decreasing pharyngeal residues in elderly patients with moderate to severe dysphagia.
Huppertz et al. (57)	OD patients in elderly nursing homes	Adding amylase resistant substance to pre-thickening ONS can increase the safety of swallowing and is suitable for OD patients.
Cuomo et al. (58)	OD patients in nursing homes	Configured by vegetable cream and protein powder blend of paste-like material for feeding, its high nutritional value, rheological properties close to commercially available preparations.
Kaae et al. (59)	OD patients caused by head and neck cancer	Gel-like polymers composed of hydroxypropyl methylcellulose K200M (HPMC) and polyethylene oxide wsr-301 (PEO) can reduce the incidence of coughing.

This table provides an overview of food-specific studies conducted by the research teams mentioned in OD patients.

Microgel is a potential substance that can change the texture characteristics of food and provide greater benefits to patients with dysphagia. In addition to its function as a texture regulator, microgels are also proposed as carriers of non-polar compounds, such as vitamins, antibacterial agents and antioxidants. These microgels are generally formed by shear gelation or prefabricated droplet gelation (60). A recent study showed that two-step heating of 0.5% microgels at 37 and 90°C could improve the physical and functional properties of a new type of surimi gel for dysphagia (61).

Since the publication of ≪National Dysphagia Diet≫ (NDD) in 2002 (105). In North America, concentrate for dysphagia treatment is divided into Thin (0–50 cP), Nectar-like (51–350 cP), Honey-like (351–1,750 cP) and spoon-like (>1,750 cP) fluids.The research team of Reyes-Torres et al. (51), in an RCT experiment named Design and Implementation of Modified-texture diet in older adults with or without swallowing, compared a structured modified food and thickening beverage diet with honey or pudding viscosity and control amount with the standard treatment group of equal calories. The results showed that the physiological indexes (body weight, grip strength and phase angle) of foods with nectar or pudding viscosity were better than those of traditional foods. In Japan, a multi-center study on the advantages of enzyme-processed and softened new foods for patients with impaired chewing and mild dysphagia showed that iEAT (a new food processed and softened by enzyme, EN Otsuka Pharmaceutical Co. Ltd., Japan) had smaller intake and did not need to be repeated swallowing compared with the traditional structurally modified diet with equal energy. This new food may be suitable for stroke, dementia, and other patients with OD (55). Kyodo et al. (56) conducted an RCT study on reducing the risk of aspiration in elderly patients with moderate to severe dysphagia by using a clean diet containing gelling agents. The study found that the concentrated soup diet containing gelling agents may help prevent aspiration in elderly patients with moderate to severe dysphagia by reducing the residue in the pharynx.Dysphagia patients also need sensory and nutritious food. So Cuomo et al. (58) came up with a new food formulation for patients with dysphagia: a cereal-based protein powder and a vegetable cream. They evaluated the nutritional and rheological properties of the two products and found that the rheological properties of the two products are equally suitable for patients with dysphagia, and that they also have a good taste and can compete with commercially available products (58). Compared with the standard paste without cementing agent, the advantages of the paste containing cementing agent are considered to be related to the decrease of adhesion (56). Another RCT study on the use of pre-thickened ONS in elderly patients with OD in nursing homes to improve OD and malnutrition found that providing a pre-thickened anti-amylase ONS is consistent with the severity of OD, which can improve the swallowing efficacy and safety and oral intake compliance of nursing home residents with OD and malnutrition (risk) (57). In principle, efforts should be made to adapt different types of thickeners to standard guidelines. This will provide greater clinical assurance and will allow for more effective management to avoid overuse of the product (62).

### The author's perspective view

OD evaluation and diagnosis are difficult. The existing have shown that dynamic fluorescence imaging and ultrasound technology are important tools for evaluating OD (63–67). In terms of food selection, according to the framework of IDDSI, the selection of food at level four can be effectively accepted by patients with dysphagia. According to the loss angle tangent principle, choosing foods with larger tanδ can be swallowed well by patients. Foods suitable for patients with dysphagia can be optimized using IDDSI as well as through viscosity, viscoelasticity (G ^*^, G00, yield stress), texture properties (cohesiveness, adhesion, gumminess) and tribological methods. The use of gums (XG, FG, MS) shows some advantages because they have better stability, solubility and viscosity properties. According to the provisions of the American Diet Association in 2002, honey or pudding viscosity (350–1,750 cP) is relatively better than traditional improved food. As Sungsinchai et al. review, foods for head and neck cancer patients should be soft and moist; these foods include soup, pudding, yogurt, jelly, applesauce, cream, gel, smoothie, soft biscuit or milk baked goods (68). At the same time, using a large number of natural capsaicin, some foods processed by enzyme process may have obvious effects. But some studies have shown that improved foods associated with OD may cause dehydration problems (69, 70). O'Keeffe expressed doubts about whether improved food could effectively prevent aspiration pneumonia in OD patients (71). Swallowing training may be an effective method. In a review of intervention and treatment of dysphagia, it is clarified that behavioral training and retraining combined with diet treatment can achieve good results (72). Therefore, under the existing evidence, the author's team recommended that patients adopt the combination of swallowing behavior training and therapeutic diet to overcome OD, so as to achieve the goal of basically normal swallowing. In addition, because the reasons for OD are diverse, clinical dietitians need to develop personalized programs when selecting appropriate foods for patients.

## Non-oral dysphagia (esophageal cancer and achalasia for example)

### Anatomical features of malignant esophageal tumors

Esophageal cancer affects more than 450,000 people worldwide and currently ranks sixth in cancer-related mortality (73). Normal adult esophagus 18–26 cm (7–8 inches) long. When expanding, the backplane of the cavity is about 2 cm (0.79 in), and the side plane is about 3 cm (1.2 in). The muscles of the upper three segments of the esophagus consist of striated muscles. The next two-thirds are composed of smooth muscle. The lower esophageal sphincter is completely composed of smooth muscle (74). The reason for dysphagia in patients with esophageal cancer is that malignant tumors grow or penetrate the esophageal wall, thus squeezing the original esophagus and causing food to fail to pass normally. Another is the gradual involvement of cancer tissue throughout the esophagus, into the lumen invasion of the corresponding muscle tissue, muscle tissue gradually lost normal function, generally manifested as reflux, etc., (75). Unlike OD, general OD patients may have cough, asphyxia, nasal reflux and tracheobronchial inhalation at the onset of dysphagia (76). But patients with esophageal dysphagia will report food sticking in the throat or upper chest after swallowing for a few seconds. It is pointed out that the location of the substernal obstruction in the upper sternum notch or behind the sternum is highly correlated with anatomical obstruction (such as ring, mass or stenosis) (64, 74). Chen et al. concluded in a study that esophageal malignant tumors were mostly located in the middle esophagus (77). Eduard Matkovic et al. mentioned in summarizing the characteristics of esophageal malignant tumors that pain generally occurs in the upper abdomen and behind the sternum (74). Such pain may affect appetite and even swallow to some extent.

In addition to being used in OD, PEG is also widely used and has unique advantages in patients with esophageal cancer and dysphagia. However, as mentioned earlier, the esophageal damage that PEG may cause is impossible to avoid, especially in patients with abnormal esophageal anatomy, but esophageal anatomic abnormalities are common in patients with esophageal malignant tumors (78).

### Pathophysiological characteristics of achalasia of the cardia

Achalasia is one of the common causes of dysphagia in the non-oral period. The pathogenesis of achalasia is still unclear. However, research suggested that the root cause of primary achalasia was the loss of inhibitory myenteric neurons in the Auerbach plexus. Achalasia is a primary esophageal motility disorder characterized by the disappearance of esophageal peristalsis and the inability to relax the lower esophageal sphincter when swallowing (79–82). The peristaltic process is very complex and depends on the precise interaction between the central nervous system, myenteric plexus and esophageal smooth muscle. Progressive delayed contraction of subesophageal muscles in the healthy esophagus can be due to inhibitory neurotransmitters (83, 84). The main cause of achalasia mentioned in the ACG clinical guidelines is the selective loss of inhibitory neurons in the distal esophageal intermuscular plexus and the lower esophageal sphincter (LES), resulting in a neuronal imbalance of excitatory and inhibitory activities. Exciting neurons release acetylcholine, while inhibitory neurons mainly release vasoactive intestinal peptides and nitric oxide (85). Reduced local vasoactive intestinal peptide and nitric oxide and non-corresponding excitatory activity lead to failure of lower esophageal sphincter relaxation and esophageal peristalsis disorder (86–88). Degenerative changes in myelinated and unmyelinated vagus nerve fibers have been demonstrated in surgical biopsy specimens from a study in the last century (89, 90). In addition, immune factors are also one of the possible causes of achalasia. However, since this paper is to develop food services that are consistent with the fluid dynamics of swallowing to the esophagus in patients with achalasia, it focuses on the related factors of muscle tissue, and immune factors are not described in detail. A retrospective cohort study showed that more than 70% of patients with achalasia had moderate/high malnutrition risk (91). Therefore, it is urgent to solve the nutritional problems of these patients.

### How to supply nutrition through non-tube feeding for patients with non-oral dysphagia

According to the previous summary, we can conclude that the main reason for dysphagia caused by esophageal cancer is that the tumor grows or penetrates the esophageal wall, resulting in food inability to swallow or cancer tissue invading muscle tissue, resulting in reflux during swallowing. The main reason for dysphagia caused by achalasia is the failure of esophageal sphincter relaxation and esophageal peristalsis disorder. Therefore, the use of appropriate thickeners and hydrocolloids to change food consistency and cohesiveness is necessary (92–94). However, too sticky food may lead to further increased obstruction. In the process of swallowing to the esophagus through the oropharynx, because such patients usually have muscle-related problems, they need to use shear stress during swallowing. The starch-based thickeners have been shown to produce strongly non-Newtonian fluids with shear-thinning properties (95, 96). Germain's research team successfully used the Hershel-Bulkley model [Eq. (5)] to describe the thickening behavior of starch. The model formula included the power-law relationship between shear stress and strain rate and small yield stress (95).


(5) $$\begin{array}{l} \tau =\tau_{0}+K\dot{\gamma^{n}} \end{array}$$

The flow behavior index (*n*) is equal to 1 for Newtonian fluids; in such cases, the consistency index (K) is equivalent to the fluid's viscosity (97). In addition, it is mentioned in a systematic review that adding xanthan gum thickeners in liquids can also produce non-Newtonian properties (93). O'Leary et al. (97) proposed that the non-Newtonian properties of liquids may make them particularly suitable for the treatment of dysphagia. It can change the properties of liquids to change their non-Newtonian reactions (flow behavior index) to optimize the therapeutic effect (87). Meshkinpour et al. found in a clinical study that viscous swallowing can cause dysphagia and esophageal peristalsis disorders (98). We also found four specific food studies for patients with non-oral dysphagia (Table 3). In the relevant literature retrieval, a study on Riceberry rice puddings attracted the author's team (99). The product in the study looked at IDDSI 3–4 levels in terms of viscosity and was also applicable to patients with high blood sugar, a feature that compensates for most food deficiencies and deficiencies. Linda Killeen's team studied a Gum-Containing Thickener, they found that the thickener was superior to starch-based thickener due to its good viscosity stability during swallowing (100). Isabelle Germain's study provides a thick beverage designated Honey (351–1,750 cP), with significant improvements in BMI and nutritional laboratory test results in elderly people with dysphagia (101). At the same time, a study of injecting botulinum toxin to reduce esophageal motility disorders has attracted our attention. This study found that for patients with primary esophageal motility disorders without achalasia, injecting botulinum toxin can alleviate the symptoms of dysphagia (102). Neurogenic loss, such as difficulty swallowing caused by the effect of thyroidectomy on the recurrent laryngeal nerve, is also clinically common. We also found that the research and development of food products suitable for esophageal dysphagia are still rare, which is one of the areas that need our research.

**Table 3:** Summary of food products suitable for Non-OD patients.

**Investigator**	**Target population**	**Results**
Suttireung et al. (99)	Dysphagia patients with hyerglycemia	Riceberry rice puddings can be effectively tolerated by patients with hyperplycemic dysphagia and enjoy eating food
Killen et al. (100)	Adult patients with dysphagia	Use of the gum-containing thickener is preferred to a starch-based thickener due to the stability of its viscosity during consumption
Germain et al. (101)	Elderly people with dysphagia and malnutrition	Fragmented or muddy food is acceptable to older people and has achieved good results

This table provides an overview of food-specific studies conducted by the research teams mentioned in Non-OD Patients.

## Conclusion and implications

Dysphagia is classified according to location and evidence of drug delivery failure due to mechanical or inflammatory processes (103). Once dysphagia occurs, the incidence of malnutrition in patients is often greatly increased. Therefore, clinical classification is needed for these patients. Solving this problem requires not only clinicians, but also food science engineers to focus on manufacturing texture-improved foods from the perspective of food fluid mechanics. At the same time, it is important to consider that the mixing of saliva and food may change its rheological properties. Although the rheological properties after mixing with saliva are rarely analyzed, this must be considered in dietary prescriptions for dysphagia (104). IDDSI is an existing guideline and simple measurement method for evaluating food hydrodynamics. When making food for patients with dysphagia, it is best to follow the IDDSI guidelines. In addition, to allow patients to adapt to texture improved food, sensory and psychological requirements are also the focus of our consideration. In the process of reviewing esophageal dysphagia, we found that there are few studies on texture-modified foods for this disease, which is also an area that scientists need to solve.

## Author contributions

AM: conceptualization and funding acquisition. ZS: methodology and writing—original draft. ZS and AH: formal analysis. YH and AM: writing—review and editing. AH: visualization. All authors contributed to the article and approved the submitted version.

## Funding

This work was supported by the Shanghai Health Medical College Seed Fund (SFP-18-21-15-002) and Xinjiang Uygur Autonomous Region Innovation Training Program Project (S202113558007).

## Conflict of interest

The authors declare that the research was conducted in the absence of any commercial or financial relationships that could be construed as a potential conflict of interest.

## Publisher's note

All claims expressed in this article are solely those of the authors and do not necessarily represent those of their affiliated organizations, or those of the publisher, the editors and the reviewers. Any product that may be evaluated in this article, or claim that may be made by its manufacturer, is not guaranteed or endorsed by the publisher.
